# Supplementary material for: Silencing the Circadian Clock Genes Cycle and Clock Disrupts Reproductive–Metabolic Homeostasis but Does Not Induce Reproductive Diapause in Arma chinensis
Source: Insects. 2025 Nov 23;16(12):1192. doi: 10.3390/insects16121192 (PMC12734136; doi:10.3390/insects16121192)

Supplementary data

Table S1. The primers used in this study.

Primer name	Forward primer sequences (5'-3')	Reverse primer sequences (5'-3')
AcCyc	GCGATGCTGCCAGTGAAA	GGAGGGGGAATATCTTGTAG
AcClk-1	TCGAGAACCTGTCCCTTTGG	TGTCCAATCCTCCCCTTTT
AcClk-2	AAAAGGGGAGGATTGGACA	CCACGGCTCTGATTTTGC
AcClk-3	GCAAAATCAGAGCCGTGG	CGCTAAAATACAAATGGTCCTC
**qRT-PCR**		
qAcCyc	GCAGAATCTCATAAAGCAGC	CTACAAGATATTCCCCCTCC
qAcClk	GGCACTTGACGGTTTCATAA	GGAAAGTGACCTAACAGGGAA
RPL27	TTCCGCGGTTGGAGTATGTG	ATTTCGGGCCAGCACTAGAA
qAcPer	GGACCTGGCAGCATTCAT	TCGGTGGAGTTCCTGAAGATGA
qAcTim	TGAAACTAAGCCAACGGAAAA	CCATAACCGCAATCTGAGG
qAcJHAMT1	TTCTGGGATAGATTTTAGGTATGGG	TTGCTGAAGCCTGGTGGTGA
qAcJHAMT2	CATAGGATGCGGGTCTG	TGGCTCTTGCGTAGTTT
qAcJHAMT3	TCCCACAAGTTGAACATAA	GAAAGAGCGGCTGAAA
qAcVg	TACCGTTGCTCACGCTGAAAGTCCA	TGGACTTTCAGCGTGAGCAACGGTA
qAcMet	GAAAGTGGACAAGACCT	GAAACAACCAATAGCAT
qAcKr-h1	CCAGCACCGCAGAAACC	CGTAAGGCGAGCAGGAA
**RNAi experiment**		
dsAcCyc	T7-TCACCACGACGGTCAAGGA	T7-GGGGCAGGAAACCAAGAAC
dsAcClk	T7-AAAAGGGGAGGATTGGACA	T7-CCACGGCTCTGATTTTGC
dsGFP	T7-CACAAGTTCAGCGTGTCCG	T7-AGTTCACCTTGATGCCGTTC

T7: (T7 promoter sequence for in vitro transcription of dsRNAs): GATCACTAATACGACTCACTATAGGG

Table S2. Information on the insects included in the phylogenetic analysis for Cycle and Clock.

Species	Unigene ID	Order
Drosophila melanogaster	NP_511126.2	Methoprene-tolerant
Drosophila melanogaster	AAC62234.1	Clock
Arma chinensis	This study	Clock
Arma chinensis	This study	Cycle
Pyrrhocoris apterus	XP_059609357.1	Clock
Anastrepha fraterculus	AQV08516.1	Clock
Anabrus simplex	XP_068082087.1	Clock
Bemisia tabaci	XP_018899499.1	Clock
Cimex lectularius	XP_014245918.1	Clock
Halyomorpha halys	XP_014282120.1	Clock
Laodelphax striatellus	RZF36018.1	Clock
Lutzomyia longipalpis	XP_055688522.1	Clock
Macrosteles quadrilineatus	XP_054274274.1	Clock
Nilaparvata lugens	XP_039284971.1	Clock
Phlebotomus argentipes	XP_059609357.1	Clock
Phlebotomus papatasi	XP_055703322.1	Clock
Riptortus pedestris	BAN20981.1	Clock
Cephus cinctus	XP_015590608.1	Clock
Chelonus insularis	XP_034935432.1	Clock
Frieseomelitta varia	XP_043518506.1	Clock
Phlebotomus argentipes	XP_059609357.1	Clock
Drosophila melanogaster	NP_524168.2	Cycle
Aethina tumida	XP_019873821.1	Cycle
Chrysoperla carnea	XP_044733956.1	Cycle
Chrysoperla nipponensis	WAM46968.1	Cycle
Cimex lectularius	XP_024083958.1	Cycle
Colletes gigas	XP_043260315.1	Cycle
Halyomorpha halys	XP_024218927.1	Cycle

Hylaeus volcanicus	XP_053974749.1	Cycle
Macrosteles quadrilineatus	XP_054274439.1	Cycle
Onthophagus taurus	XP_022917457.1	Cycle
Schistocerca americana	XP_046997855.1	Cycle
Schistocerca cancellata	XP_049784787.1	Cycle
Schistocerca gregaria	XP_049827997.1	Cycle
Schistocerca nitens	XP_049811755.1	Cycle
Tenebrio molitor	XP_068918134.1	Cycle
Tribolium castaneum	XP_064212799.1	Cycle

Figure S1 The potential off-targets of dsAcCyc and dsAcClk were evaluated using "dsRIP". (A) All off-targets for dsAcClk. (B) All off-targets for dsAcCyc.

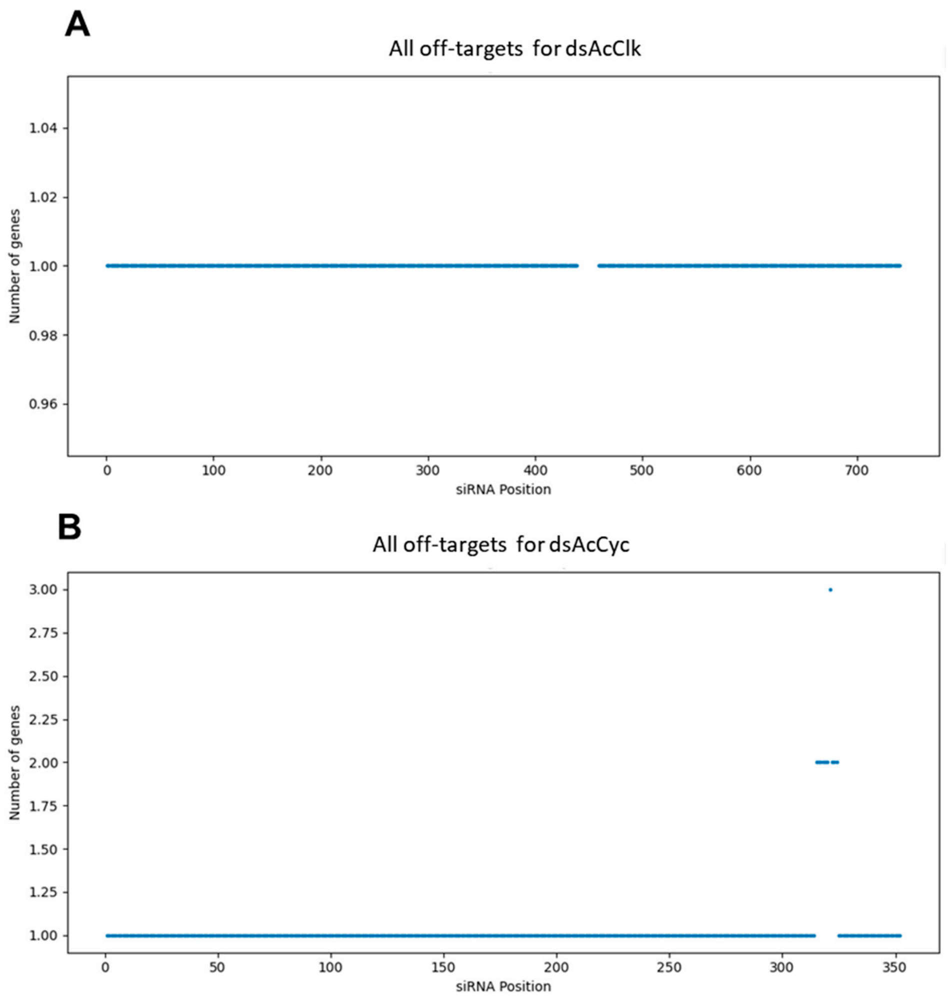

Supplement: Supplementary file 1 [file insects-16-01192-s001.zip › Supplementary data.pdf]
